# Improved fruit fly algorithm on structural optimization

**DOI:** 10.1186/s40708-020-0102-9

**Published:** 2020-02-16

**Authors:** Yancang Li, Muxuan Han

**Affiliations:** 1 College of Water Conservancy and Hydroelectric Power, Hebei University of Engineering, Handan, Heibei Province China; 2 College of Civil Engineering, Hebei University of Engineering, Handan, Heibei Province China

**Keywords:** Truss structure optimization, Fruit fly algorithm, Improvement, Immune response

## Abstract

To improve the efficiency of the structural optimization design in truss calculation, an improved fruit fly optimization algorithm was proposed for truss structure optimization. The fruit fly optimization algorithm was a novel swarm intelligence algorithm. In the standard fruit fly optimization algorithm, it is difficult to solve the high-dimensional nonlinear optimization problem and easy to fall into the local optimum. To overcome the shortcomings of the basic fruit fly optimization algorithm, the immune algorithm self–non-self antigen recognition mechanism and the immune system learn–memory–forgetting knowledge processing mechanism were employed. The improved algorithm was introduced to the structural optimization. Optimization results and comparison with other algorithms show that the stability of improved fruit fly optimization algorithm is apparently improved and the efficiency is obviously remarkable. This study provides a more effective solution to structural optimization problems.

## Introduction

With the rapid development of computer technology, the efficiency of structural optimization is greatly improved, and structural designers can have more time and energy to consider how to get better structural design scheme. In 1974, Schimit and Farshi proposed to combine finite element theory with mathematical induction theory to solve the optimal weight problem of engineering structure. Structural optimization has stepped into a new era [1]. After that, the intelligent optimization algorithm is also widely applied to the structural optimization, and the modern structural optimization method is gradually applied to the engineering practice. Now, after years of research and development, structural optimization has changed from the original optimization of structure size to the present optimization of topology and further optimization of material distribution. From a single objective optimization problem, multiple objectives are optimized simultaneously. Azamirad and Arezoo [2] proposed an improved software package for stamping die structure that can greatly reduce the weight. Ide [3] designed the lightweight structure with the structural optimization method, and successfully realized the lightweight gear box design by using the design method of reducing contact constraint stress. Kaveh [4] proposed water evaporation optimization algorithm (WEO), which is a population-based intelligent optimization algorithm inspired by physics and used for continuous structural optimization. In order to apply different optimization solvers to various finite-based structural topology optimization problems Rojas-Laband [5], developed a widely representative example library of mechanism design problems with minimum compliance, minimum volume and different sizes. Sivapuram [6] discussed various calculus methods and numerical methods commonly used to solve structural topology optimization problems.

Inspired by the foraging process of fruit flies, scholar Pan [7] proposed a more efficient swarm intelligence optimization algorithm in 2012: fruit fly optimization algorithm (FOA). The algorithm has the advantages of clear principle, fewer parameters and simple operation. However, there are also some shortcomings, such as weak ability to solve complex, high-dimensional and nonlinear optimization problems and easy to fall into local extremum. In view of the above typical problems, many scholars have proposed many improvements. As for the generation of candidate solutions [8], added an escape parameter that can be negative to the taste concentration judgment value, so that the candidate solution can take a negative value. A novel double strategy evolutionary fruit fly optimization algorithm (DSEFOA) was proposed by Fang [9]. DSEFOA dynamically divided the fruit fly population into spermatogonium subgroups and ordinary subgroups, and adopted different strategies to update the evolution of drosophila at different levels of evolution, which improved the optimization ability of the whole population. In terms of search radius, Sang et al. [10] introduced adaptive parameters to adjust the search radius of drosophila so as to better balance the global search capability and local search capability. Cao [11] proposed to replace the traditional search mechanism with sector search mechanism, and the new fruit fly algorithm was generated to effectively improve the stability of the algorithm. In terms of flight strategy [12–14], added the operation of group collaboration and random perturbation to solve the problem of premature development of the algorithm. Sheng [15] proposed that the length of fruit fly search for Uber should be dynamically changed according to the change rate of concentration difference, which can effectively balance the global optimization ability and the local optimization ability. In terms of population diversity [16], divided the drosophila population into multiple subpopulations of the same size. Han [17] et al. proposed a dynamic twin group co-evolutionary FOA to improve the search accuracy. Xin [18] used the gaussian sampling method to update the fruit fly. This method can increase the chance of jumping out of the local extremum in the early stage of the algorithm and conduct more accurate search in the later stage.

Aiming at improving the performance of the standard fruit fly optimization algorithm, the hybrid algorithm was designed to combine with fusion immune response. Immune algorithm was fully utilized to improve the deficiency of fruit fly algorithm that is prone to fall into local extreme value in the later stage. In other words, when the number of evolutionary stasis steps *t* is greater than the threshold value of evolutionary stasis steps *T*, immune operation is performed to overcome the defect of basic fruit fly algorithm. The improved algorithm was proved to have better robustness and intelligence through standard functions and tests for solving 0–1 knapsack problems [19–21]. Finally, it was applied to the optimization of truss structure [22] and compared with other algorithms to verify the feasibility of the improvements.

## Basic fruit fly algorithm

**Fig. 1:**
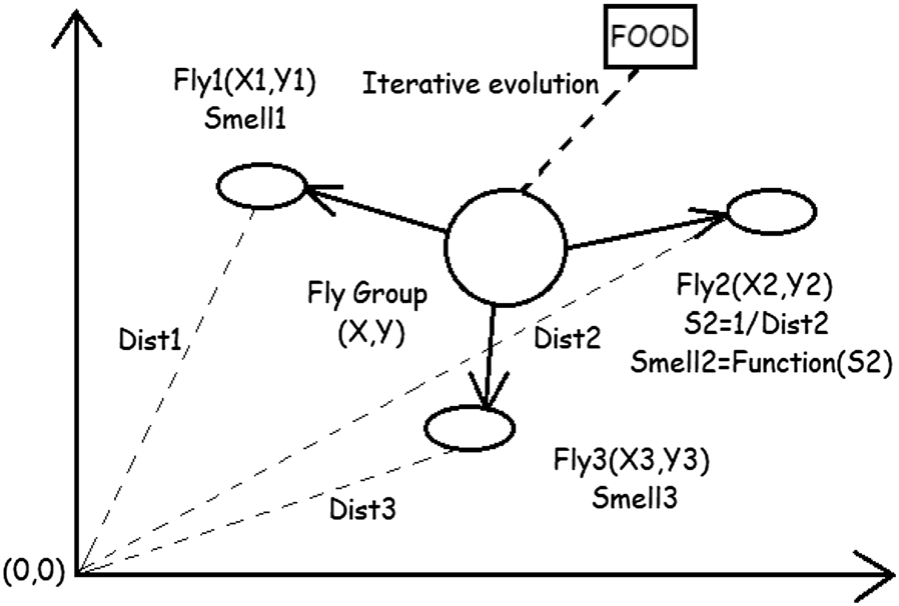
FOA foraging schematic diagram

FOA (fruit fly optimization algorithm) is a new heuristic algorithm that simulates the foraging activities of fruit flies in nature to seek the optimal solution of the objective function. The foraging iteration diagram of fruit flies is shown in Fig. 1.

The basic steps are as follows [23–26]:

- Step 1: initialize parameters. Set *Sizepop* and *Maxgen* of the population size, and initialize the population position:
  1 $$ \left( {X_{{{\text{\_axis}}}} , Y_{{\_{\text{axis}}}} } \right). $$
- Step 2: fruit fly searches in the olfactory system, which can make the search direction and the search step randomly. Random value (RV) is to be the search distance, and the position of the population is updated simultaneously:
  2 $$ \left\{ {\begin{array}{*{20}c} {X_{i} = X_{{\_{\text{axis}}}} + {\text{RV}}} \\ {Y_{i} = Y_{{\_{\text{axis}}}} + {\text{RV}}} \\ \end{array} } \right.. $$
- Step 3: since the exact location of the food is unknown, it is necessary to calculate the distance (${\text{Dist}}_{i}$) between the fruit flies and the origin of the coordinate and then calculate the taste concentration parameter ($S_{i}$):
  3 $$ {\text{Dist}}_{i} = \sqrt {X_{i}^{2} + Y_{i}^{2} } $$
  4 $$ S_{i} = \frac{1}{{{\text{Dist}}_{i} }}. $$
- Step 4: substitute the fruit fly flavor concentration determination value (*S*_*i*_) into the taste concentration decision function, the fitness function, then we will obtain the individual taste concentration of the fruit flies ${\text{Smell}}_{i}$.
  5 $$ {\text{Smell}}_{i} = {\text{Fitness}}\left( {S_{i} } \right). $$
- Step 5: identify the individual with the highest flavor concentration in the drosophila population.
  6 $$ \left[ {{\text{bestSmell}},{\text{bestIndex}}} \right] = { \hbox{min} }\left( {\text{Smell}} \right). $$
- Step 6: retain the best flavor concentration value and coordinate, and other individuals in the population fly to this position:
  7 $$ {\text{SmellBest}} = {\text{bestSmell}}. $$
  8 $$ \left\{ {\begin{array}{*{20}c} {X_{{\_{\text{axis}}}} = X\left( {\text{bestIndex}} \right)} \\ {Y_{{\_{\text{axis}}}} = Y\left( {\text{bestIndex}} \right)} \\ \end{array} } \right.. $$
- Step 7: termination condition, judge whether the concentration of the best position is better than that of the previous generation, and reach the maximum number of iterations; otherwise, skip step 2 to enter the iterative optimization.

## Improved fruit fly algorithm with immune response

Since fruit fly has a relatively developed olfactory and visual system, it first carries out a wide range of search through the sense of smell and sends out food odor information to the surrounding drosophila in the process of foraging. When a fly is found to have a higher concentration by comparison, the individual flies will rely on visual function to fly to that location [27, 28]. It is precisely because of such population characteristics that the diversity of the population is reduced, the algorithm that simulates its characteristics, like other bionic intelligent algorithms, has the defect that it is easy to fall into local optimization, which leads to the problem of early maturity [29].

The immune algorithm was inspired by somatic cell theory and network theory [30]. And it can realize the function of self-regulation by generating different antibodies similar to the immune system [31]. This algorithm has strong local search ability. By using this, we can introduce it into the later stage of fruit fly algorithm execution to improve the basic fruit fly algorithm. The new algorithm IAFOA can be used to balance the deficiency of fruit fly algorithm that is prone to fall into local optimal, and improve the search efficiency.

### Immune algorithm

Immune algorithm (IA) is a kind of bionic optimization algorithm. In 1990, Bersini [32] first used immune algorithm to solve problems. By simulating biological immune system identify antigen (objective function), simulation of the principle of the memory in the immune system, combination of antigen and antibody (optimization) solution, and diversity of imitation immune system, IA algorithm can realize the antigen recognition, cell differentiation, and memory of the immune system and self-regulating function [2, 33, 34]. The basic steps of IA are as follows:

- Step 1: antigen recognition. Input objective function and constraint conditions as antigen of immune algorithm;
- Step 2: generate the initial antibody. Generate initial antibodies randomly within the solution space;
- Step 3: calculate the compatibility (fitness evaluation). According to the given fitness evaluation function, the affinities between antibodies and antigens and between antibodies and antibodies were determined. The compatibility between antigen and antibody $A_{v}$ is defined as follows:
  9 $$ A_{v} = 1/\left[ {1 + O_{{Pt_{v} }} } \right] , $$
  where $O_{{Pt_{v} }}$ represents the matching degree of antigen and antibody, and the value of $A_{v}$ is between 0 and 1. When $O_{{Pt_{v} }} = 0$, $A_{v} = 1$, indicating that the antibody matches the antigen very well, that is, the antibody is the optimal solution.
- Step 4: update memory units. The antibody with the highest affinity to antigen calculated in step 3 was added into the memory unit and replaced by the original antibody.
- Step 5: promote and inhibit node production. Calculate the expected value $E_{xi}$ of antibody *i*, the low expected value of antibody will be suppressed.
  10 $$ E_{xi} = A_{i} /C_{i}, $$
  where $A_{i}$ is the affinity between antigen and antibody *i*, and $C_{i}$ is the number of antibody *i*;
- Step 6: generate new antibodies. The father generation produces the next generation antibody through heredity, mutation and crossover.
- Step 7: whether termination conditions are met. Yes, stop the algorithm; No, skip to step 3.

### Population diversity improvement of IAFOA

The immune system is the basic defense system to maintain the normal metabolism of living organisms by blocking the invasion of bacteria [27]. The system recognizes gene types to produce different antibodies, promotes the emergence of new individuals and inhibits the excessive production of individuals through regulatory mechanisms, so as to achieve biological diversity [4].

**Fig. 2:**
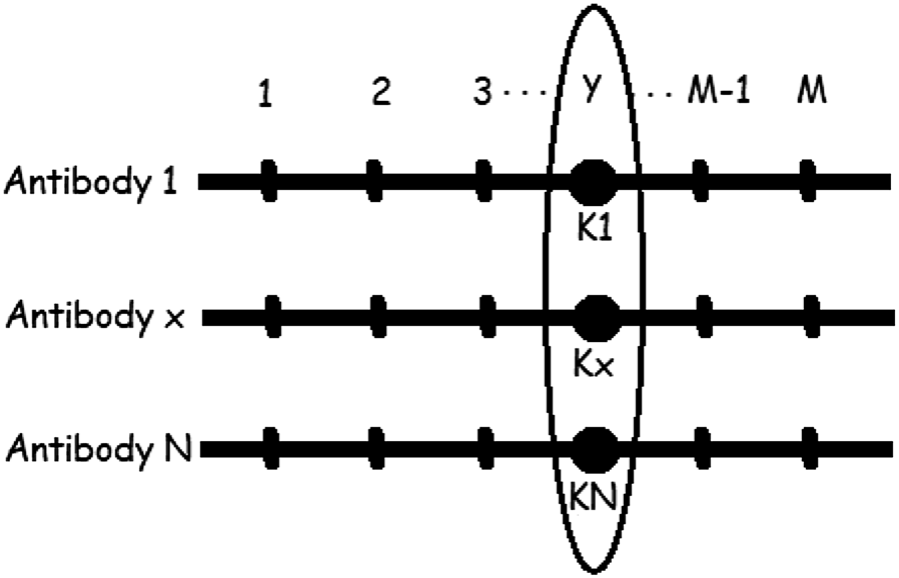
Information entropy of genes

Suppose the immune system has *N* antibodies, and each antibody has *M* genes, as shown in Fig. 2. The information entropy $H_{j} \left( N \right)$ of the *j* gene is:

11 $$ H_{j} \left( N \right) = \mathop \sum \limits_{i = 1}^{N} \left( { - P_{ij} { \log }P_{ij} } \right). $$

If all the alleles of the antibody are the same at position *j*, then $H_{j} \left( N \right)$ is equal to 0.

Therefore, the average information entropy $H\left( N \right)$ of the system is as follows:

12 $$ H\left( N \right) = \mathop \sum \limits_{j = 1}^{M} H_{j} \left( N \right)/M . $$

### The implementation of IAFOA

Based on the standard fruit fly algorithm, FOA algorithm was first used to construct the feasible solution set. Then, the number of evolutionary stasis steps *t* was used as the trigger condition [35]. When *t *>* T* (threshold of evolutionary stasis steps), the IA search process was invoked, and the process of immune factors in the immune algorithm (corresponding to individual drosophila) seeking antigens (corresponding to food source in the drosophila algorithm) producing antibodies (optimal solution) was used to expand the search space. The elite retention strategy was carried out in the feasible solution set obtained after IA search. And the obtained optimization solution was used together with the original feasible solution set to update the concentration of odor information in the search space, so as to guide other fruit fly’s path search mechanism.

The threshold value of evolutionary stagnation step *T* is the index to enter the IA algorithm. The premature introduction of IA algorithm is not conducive to the search ability of IA, or even the convergence of feasible solutions. Through multiple independent experiments, *T *= *6* was adopted as the trigger value for entering IA algorithm.

If the trigger condition is satisfied, a fixed immune factor redistribution probability $P^{*}$ was employed to the optimization of space. And the different individuals were given different adaptive immune probability $P\left( i \right)$ according to their fitness values:

13 $$ P\left( i \right) = \frac{{\left( {{\text{Smellbest}} - {\text{Smell}}\left( i \right)} \right)}}{{\left( {\left( {{\text{Smellbest}} - {\text{Smellworst}}} \right)P^{*} } \right)}} $$

14 $$ \left\{ {\begin{array}{*{20}c} {{\text{if}}\left( {{\text{bestSmell}} < {\text{Smellbest}}} \right)} \\ {{\text{Smellbest}} = {\text{bestSmell}}1} \\ {{\text{else}}\left( {t = t + 1} \right)} \\ {{\text{Smellbest}} = {\text{bestSmell}}2} \\ {\text{end}} \\ \end{array} } \right., $$

where in order to avoid the algorithm falling into the local optimal solution, the initial probability of immune factor in IA algorithm was given $P^{*} = 0.25$ to randomly allocate it and increase the diversity of solutions.

The process of IAFOA is as follows:

- Step 1: initialize parameters. Set *Sizepop* and *Maxgen* of population size, initialize population positions $X_{{\_{\text{axis}}}}$ and $Y_{{\_{\text{axis}}}}$, and the number of evolutionary stagnation steps *t *= 0;
- Step 2: randomly generate fruit fly population according to Eq. (3);
- Step 3: use Eqs. (4)–(7) to operate the population;
- Step 4: record and retain the best flavor concentration value according to Eq. (14), and update the evolutionary iteration step number t;
- Step 5: judge whether *t *< *T* is true, if directly go to step 7; otherwise, according to Eq. (13), the adaptive immunity probability of individual fruit fly was calculated, and the immune operation was carried out according to the immune algorithm. For individuals who did not perform the immune operation, step 7 was taken.
- Step 6: repeat steps 2–4 for iterative search of the new population obtained by immunization;
- Step 7: set *gen *= *gen *+ 1; if *gen *< *Maxgen*, go to step 2; otherwise terminate the iteration.

**Fig. 3:**
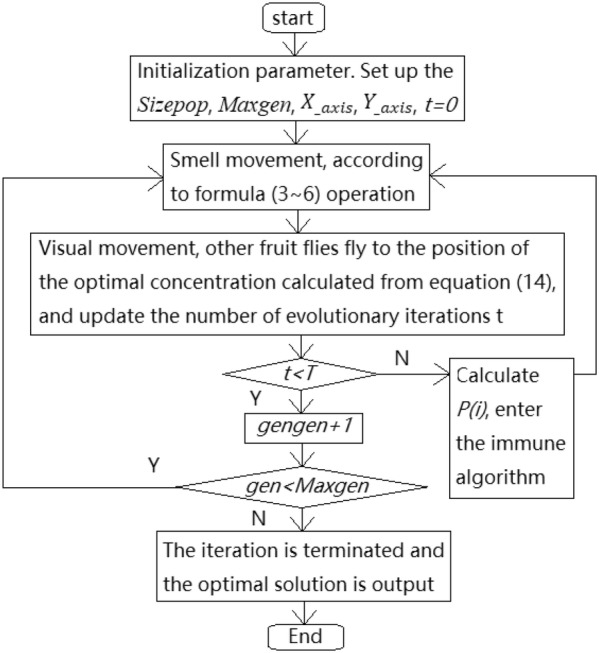
IAFOA algorithm iteration diagram

IAFOA flowchart is shown in Fig. 3.

## Simulation results

In order to verify the effectiveness of the improved algorithm, four standard test functions [5, 36] were selected for numerical simulation test to test the performance of IAFOA algorithm. In addition, 0–1 knapsack problem was selected for simulation experiment [37], and the results are compared with other algorithms. Initialization parameters: population size *Sizepop *= 200, max iteration number *Maxgen *= 1000, *dimension D *= 30, P* = 0.25, *T *= 6.

### Standard function test

The first standard function: Rosenbrock function:

15 $$ f_{1} (x) = \mathop \sum \limits_{i = 1}^{d - 1} \left[100\left({x_{i + 1} - x_{i}^{2} } \right)^{2} + ({x_{i} - {1} )^{2} } \right] . $$

The second standard function: Ackley function:

16 $$ f_{2} \left( x \right) = - a \;{ \exp }\left( { - b\sqrt {\frac{1}{d}\mathop \sum \limits_{i = 1}^{d} x_{i}^{2} } } \right) - { \exp }\left( {\frac{1}{d}\mathop \sum \limits_{i = 1}^{d} \cos (cx_{i} )} \right) + {\text{a}} + \exp \left( 1 \right). $$

The third standard function: Cross-in-tray function:

17 $$ f_{3} \left( x \right) = - 0.0001\left( {\left| {\sin \left( {x_{1} } \right)\sin \left( {x_{2} } \right)\exp \left( {\left| {100 - \frac{{\sqrt {x_{1}^{2} x_{2}^{2} } }}{\pi }} \right|} \right)} \right| + 1} \right)^{0.1} . $$

The fourth standard function: Levy function:

18 $$ f_{4} \left( x \right) = { \sin }^{2} \left( {\pi \omega_{1} } \right)   + \mathop \sum \limits_{i = 1}^{d - 1} \left( {\omega_{i} - 1} \right)^{2} \left[ {1 + 10{ \sin }^{2} \left( {\pi \omega_{i} + 1} \right)} \right] + \left( {\omega_{d} + 1} \right)^{2} \left[ {1 + { \sin }^{2} \left( {2\pi \omega_{d} } \right)} \right] , \quad {\text{where}} \;\omega_{i} = 1 + \frac{{x_{i} - 1}}{4} ,\quad {\text{for}}\; {\text{all}}\; i = 1,2, \ldots ,d. $$

**Fig. 4:**
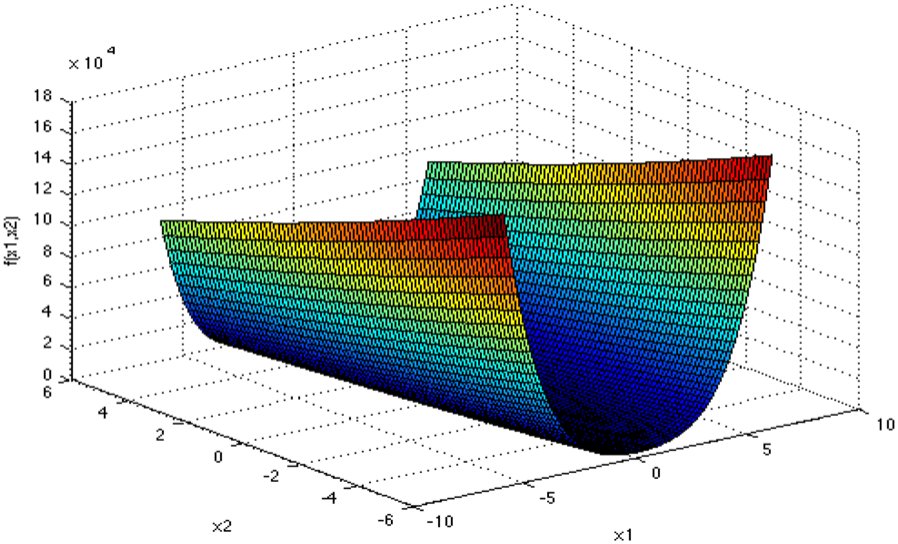
Rosenbrock function graph

**Fig. 5:**
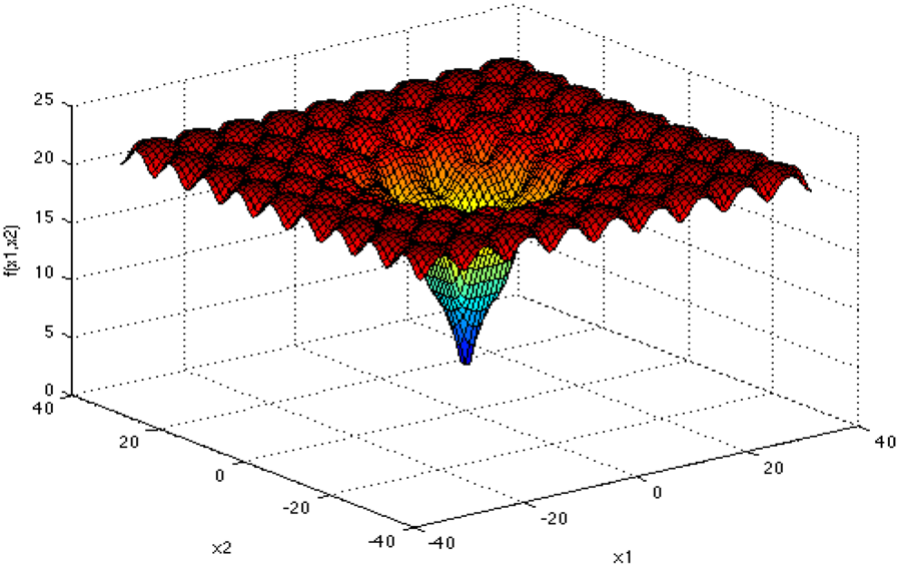
Ackley function graph

**Fig. 6:**
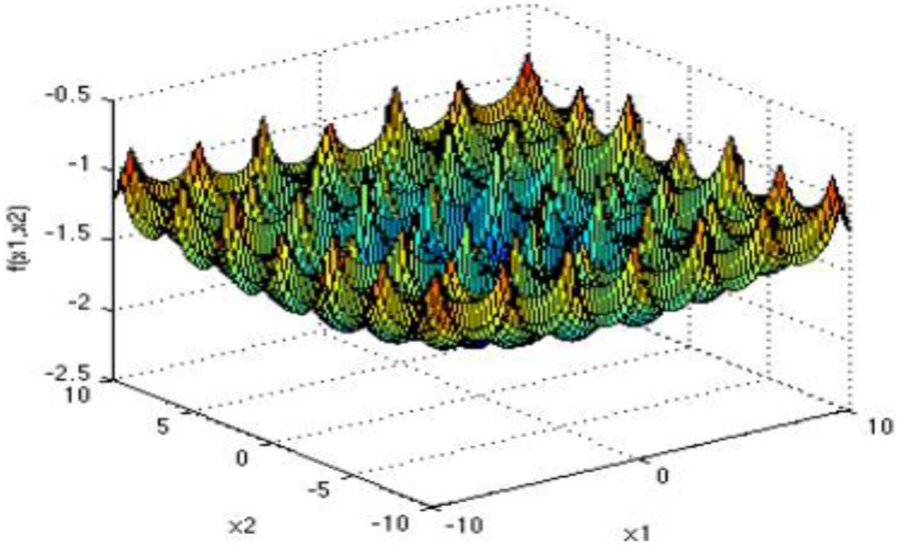
Cross-in-tray function graph

**Fig. 7:**
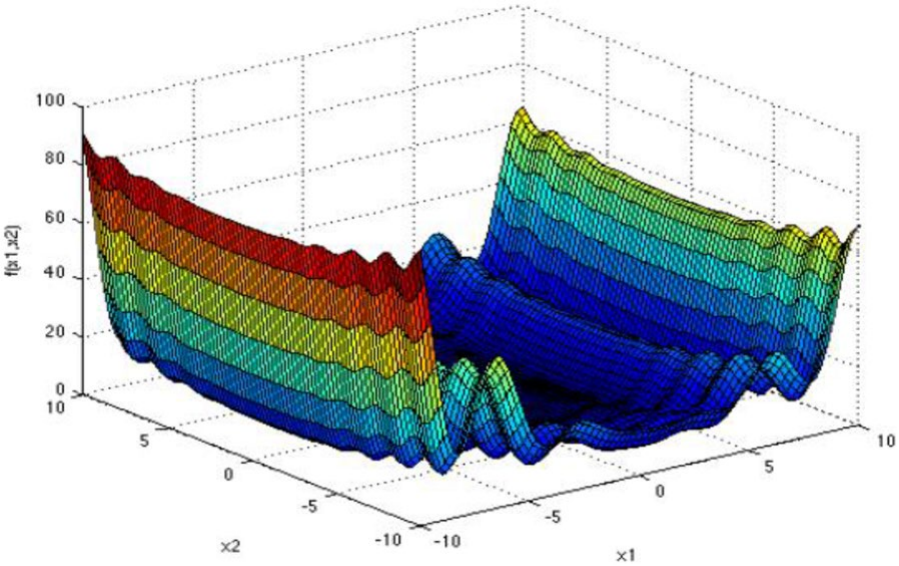
Levy function graph

Figures 4, 5, 6, and 7 show the graphs of the four functions, where Rosenbrock function is a single peak function, which is mainly used to test the convergence performance of the improved algorithm in the process of operation. Both Ackley function and Cross-in-tray function are complex multi-peak functions, which tend to make the algorithm fall into local optimization, so that the real optimal value cannot be obtained, which is used to test the ability of the improved algorithm to deal with falling into “premature”. The Levy function has a complex spatial property and is used to test the computational accuracy, convergence stability and time complexity of the improved algorithm. The selected functions and their expressions are shown in Table 1.

**Table 1:** Comparison of optimization performance for benchmark functions

Function	Dimension	Algorithm	Optimal value	Average value	Standard deviation	Running time/s
$f_{1}$	30	IA	37.1543	61.3698	33.1659	5.5329
		PSO	16.5998	34.6729	29.5267	3.9532
		FOA	15.2864	20.9341	19.5582	1.0299
		IAFOA	14.0958	17.9574	6.46931	1.3621
$f_{2}$	30	IA	2.2763	3.5297	3.061e−03	3.5146
		PSO	2.0051	2.1193	2.796e−03	4.2287
		FOA	1.6849	1.9678	3.909e−04	1.9652
		IAFOA	0	1.0052	6.9768e−03	0.9537
$f_{3}$	30	IA	2.18e−02	4.485e−02	3.0702e−01	4.7798
		PSO	1.96e−02	2.073e−01	2.785e−01	3.5669
		FOA	1.854e−01	1.6941e−01	1.1185e−01	1.2463
		IAFOA	0	0	0	0.6805
$f_{4}$	30	IA	1.952e−04	2.327e−04	6.001e−01	3.534
		PSO	1.439e−02	2.557e−02	5.026e−01	4.371
		FOA	2.005e−02	1.363e−01	3.778e−02	0.988
		IAFOA	3.958e−03	1.564e−05	2.563e−02	1.015

Four different standard functions were used to test the convergence performance, operation efficiency, handling of “local optimal” and “premature” problems, and the optimal value and average value in the optimization test. The standard deviation reflects the robustness of the algorithm. The running time reflects the convergence speed and accuracy of the algorithm. It can be seen from the calculation results in Table 2 that the fusion immune response hybrid fruit fly algorithm performs better than the standard fruit fly algorithm in different standard functions. The improved algorithm is feasible and effective.

**Table 2:** Result comparison table of TSP problem

Algorithm	Oliver30		Att48		Eil51	
	Best	Avg	Best	Avg	Best	Avg
IA	459.9	485.1	394,025	38,732	564.8	579.4
PSO	527.3	572.3	499,758	52,277	570.9	610.2
FOA	415.2	485.6	35,947	44,620	453.6	557.9
IAFOA	415.0	475.9	35,098	43,082	450.9	544.1

**Fig. 8:**
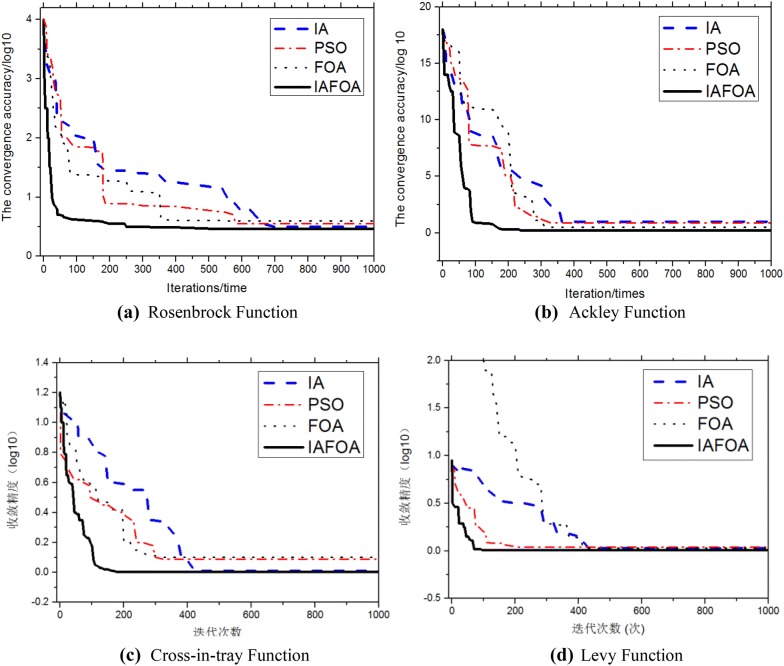
The iterative curve of the algorithm under four functions. **a** Rosenbrock function. **b** Ackley function. **c** Cross-in-tray function. **d** Levy function

In order to more intuitively see the effectiveness of improved algorithm performance, Fig. 8 shows immune algorithm (IA), particle swarm optimization (PSO), standard fly optimization algorithm. FOA) and fruit fly optimization algorithm based on immune algorithm (IAFOA) under the above four standard functions. The average value of multiple runs was used as the final result to avoid accidental errors.

It can be seen from the iterative curve of Fig. 5 that the algorithm can search the local optimal solution, in addition it reflects high convergence speed and convergence accuracy, especially at the initial stage of iterative algorithm. Take (a) in Fig. 5 as an example, when the algorithm runs about 78 times, it can jump out of the local optimal solution. When the number of iterations is about 255 times, the optimization result is generally stable. It is obviously that the improved algorithm proposed here is superior to the IA, PSO, and standard FOA.

### Solve the TSP problem

Traveling salesman problem (TSP) is a classical combinatorial optimization problem, which is a typical NP hard problem. It has important research value in the field of logistics distribution and vehicle route planning.

The TSP problem can be described as: we know the coordinate position of *N* cities or the distance between two cities. A salesman needs to go to *N* cities to sell goods. To start from one city, salesman must pass through other cities only once, and then return to the starting city. Finding out the shortest path for saleman. The mathematical model is as follows.

If $S = \left( {P_{1} ,P_{2} , \ldots ,P_{N} } \right)$ can satisfy the minimum value of $f\left( S \right)$, then access order *S* is the optimal solution of TSP problem under the condition of *N* cities, that is, the optimal path [38].

19 $$ f\left( S \right) = \mathop \sum \limits_{n = 1}^{N - 1} \left( {d\left( {P_{i} ,P_{i + 1} } \right)} \right) + d\left( {P_{N} ,P_{1} } \right), $$

where $P_{i}$ is the city code, $i \in \left( {1,2, \ldots ,N} \right)$; $d\left( {P_{i} ,P_{j} } \right)$ is the distance between city $P_{i}$ and city $P_{j}$. If only the city coordinates are known, then:

20 $$ d\left( {P_{i} ,P_{j} } \right) = \sqrt {\left( {X_{i} - X_{j} } \right)^{2} + \left( {Y_{i} - Y_{j} } \right)^{2} } , $$

where $\left( {X_{i} - X_{j} } \right)$ is the coordinate position of $P_{i}$;$d\left( {P_{i} ,P_{j} } \right) = d\left( {P_{j} ,P_{i} } \right)$.

In order to verify the performance of the IFOA in solving the TSP problem, this paper selected Oliver30, Att48 and Eil51 standard examples from the international TSP database (The Library of TSP, TSPLIB) for parameter testing. The result is shown in Table 2.

The above analysis results show that IAFOA can not only effectively solve the TSP problem, but also has a higher robustness than other algorithms.

## Optimization of truss structure by IAFOA

### Optimization model of truss structure

1. The optimization model
  The optimization model problem of truss with sectional area as the design variable is described as follows [6]:
  21 $$ { \hbox{min} }F = W\left( x \right) $$
  22 $$ {\text{s}} . {\text{t}} .   {\text{g}}_{i} \left( x \right) \le 0,\quad i = 1,2, \ldots ,m ,$$
  where ${\text{g}}_{i} \left( x \right)$ is the constraint function; *m* is the number of constraints.
2. The objective function
  23 $$ W\left( A \right) = \mathop \sum \limits_{i = 1}^{n} \rho A_{i} L_{i} ,$$
  where $W\left( A \right)$ is the weight of the structure; $A_{i}$ and $L_{i}$ are, respectively, the cross-sectional area and length of the *i*th root. $\rho$ is the density of the material; *n* is the number of design variables.
3. The constraint

Each member of the structure shall meet the requirements of strength, stiffness, stability and section size:

24 $$ \frac{{\sigma_{i} }}{\left[ \sigma \right]} - 1 \le 0, $$

25 $$ \frac{{\mu_{j} }}{{\mu_{ \hbox{max} } }} - 1 \le 0, $$

26 $$ {\text{A}}_{ \hbox{min} } \le {\text{A}}_{\text{i}} \le {\text{A}}_{ \hbox{max} } , $$

where $\sigma_{i}$ is the normal stress of the *i*th root element. $\left[ \sigma \right]$ is the allowable stress of the material. $\mu_{j}$ represents the displacement of node *j*; allowable displacement of $\mu_{ \hbox{max} }$ node *j*; ${\text{A}}_{ \text{min} }$ and ${\text{A}}_{ \text{max} }$ are the upper and lower limits of the member section, respectively.

#### Example 1

**Fig. 9:**
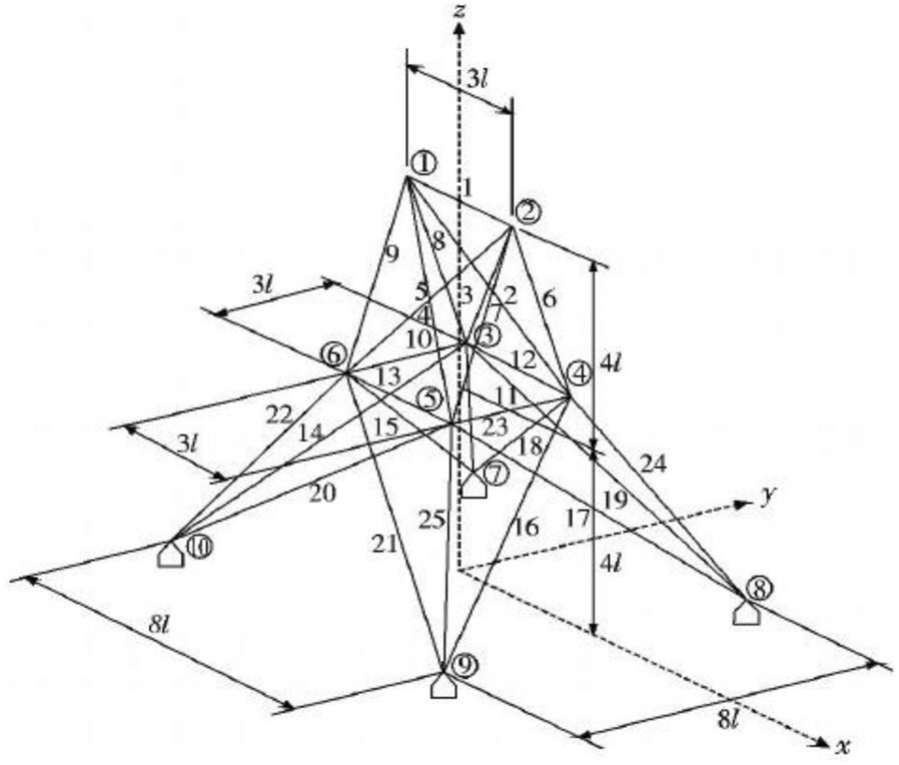
The 25-bar spatial structure

The 25-bar truss structure model [39] is established as shown in Fig. 9 and Table 3. Basic parameters of truss structure: rod length *L *= 0.635 m, elastic modulus $E = 6.895 \times 10^{4} \;{\text{MPa}}$, material density $\rho = 2.678 \times 10^{3} \;{\text{kg}}/{\text{m}}^{3}$, allowable stress range: [− 275.8275.8], maximum vertical displacement $y = 8.889\;{\text{mm}}$ of nodes 1 and 2.

**Table 3:** Load conditions of 25-bar truss structure

Node number	*F*_*x*_	*F*_y_	*F*_*z*_
1	4.448	44.482	− 22.241
2	0	44.482	− 22.241
3	22.241	0	0
6	22.241	0	0

**Fig. 10:**
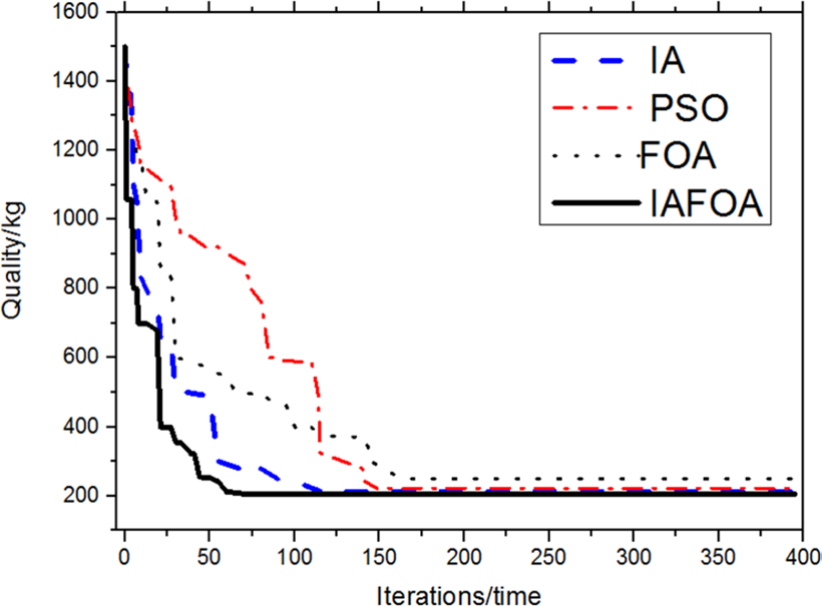
The optimization iteration curve of Example 1

The results are shown in Table 4 and Fig. 10. Under the same constraint conditions, IAFOA was used to optimize the 25-bar truss structure, and the total mass of the optimized structure was 206.591 kg. Compared to IA, mass decreased (246.436–206.591)/206.591 = 1.93%. Compared with PSO, the quality decreased (216.339–206.591)/206.591 = 4.72%; compared with FOA, the optimization results were optimized (214.702–206.591)/206.591 = 3.93%. After about 68 iterations, IAFOA can find the global optimal solution. IA searched for the global optimal solution for about 130 times, PSO for about 150 times and FOA for about 165 times. For the quality problem of global optimal solution, IAFOA is superior to the other three optimization algorithms. It is proved that IAFOA is stable in the face of complex optimization problems in the optimization process, and it is not easy to fall into the local optimal solution. Thus, IAFOA is effective.

**Table 4:** Comparison of optimization results of 25-bar truss structure

Bar number	Bar section area (unit: mm^2^)			
	IA	PSO	FOA	IAFOA
*A*_1_	65.7	64.516	64.9	64.1
*A*_2_–*A*_5_	242.6	228.5	234.5	226.3
*0*_6_–*A*_9_	2287.5	2237.6	2230.1	2240.4
*A*_10_–*A*_11_	65.12	64.516	63.149	63.56
*A*_12_–*A*_13_	1245.8	1227.9	1226.1	1223.5
*A*_14_–*A*_17_	505.1	506.9	501.7	500.9
*A*_18_–*A*_21_	92.1	83.9	89.5	85.7
*A*_22_–*A*_25_	2523.4	2575.7	2568.3	2495.6
Total weight (unit kg)	246.436	216.339	214.702	206.591

#### Example 2

**Fig. 11:**
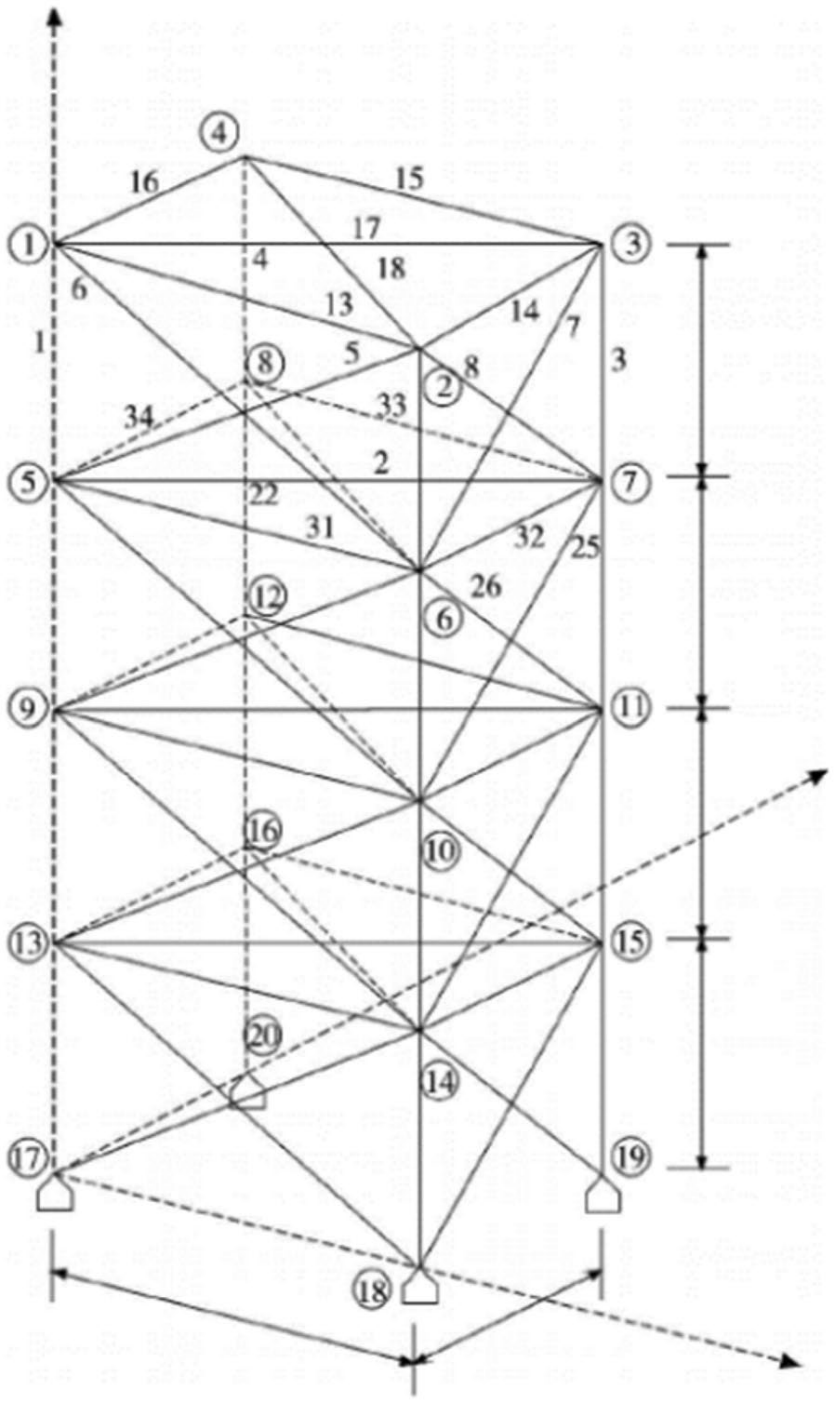
The 72-bar spatial structure

The 72-bar truss structure model [39] is established as shown in Fig. 11. 72 bars are divided into 16 groups, and the grouping condition is shown in Table 5. Material density is $\rho = 2.678 \times 10^{3} \;{\text{kg}}/{\text{m}}^{3}$, elastic modulus is $E = 6.895 \times 10^{4} \;{\text{MPa}}$, and the maximum displacement of each bar in all directions cannot exceed 6.35 mm, and maximum allowable stress is [− 172.375, 172.375], and the optimized results are shown in Table 6.

**Table 5:** The classification of 72-bar truss structure

Group number	Bar number	Group number	Bar number
*A*_1_	1, 2, 3, 4	*A*_9_	37, 38, 39, 40
*A*_2_	5, 6, 7, 8, 9, 10, 12	*A*_10_	41, 42, 43, 44, 45, 46, 47, 48
*A*_3_	13, 14, 15, 16	*A*_11_	49, 50, 51, 52
*A*_4_	17, 18	*A*_12_	53, 54
*A*_5_	19, 20, 21, 22	*A*_13_	55, 56, 57, 58
*A*_6_	23, 24, 25, 26, 27, 28, 29, 30	*A*_14_	59, 60, 61, 62, 63, 64, 65, 66
*A*_7_	31, 32, 33, 34	*A*_15_	67, 68, 69, 70
*A*_8_	35, 36	*A*_16_	71, 72

**Table 6:** Comparison of optimal designs for the 72-bar spatial truss structure

Bar group number	Bar section area (unit: mm^2^)			
	IA	PSO	FOA	IAFOA
*A*_1_	103.75	100.69	102.26	101.31
*A*_2_	358.36	359.28	372.97	349.15
*A*_3_	271.02	270.31	220.27	238.97
*A*_4_	367.88	368.96	392.01	398.29
*A*_5_	343.59	342.99	170.51	173.49
*A*_6_	337.23	337.12	353.46	336.89
*A*_7_	64.518	64.518	64.528	64.518
*A*_8_	64.518	64.518	64.518	64.518
*A*_9_	871.37	871.01	713.98	825.37
*A*_10_	318.96	318.55	373.81	332.31
*A*_11_	64.518	64.518	64.518	64.518
*A*_12_	64.518	64.518	64.518	64.518
*A*_13_	1188.59	1187.96	1330.02	1152.32
*A*_14_	324.97	325.72	324.89	332.61
*A*_15_	64.518	64.518	64.518	64.518
*A*_16_	64.518	64.518	64.518	64.518
Weight (kg)	175.03	173.87	176.31	171.98

**Fig. 12:**
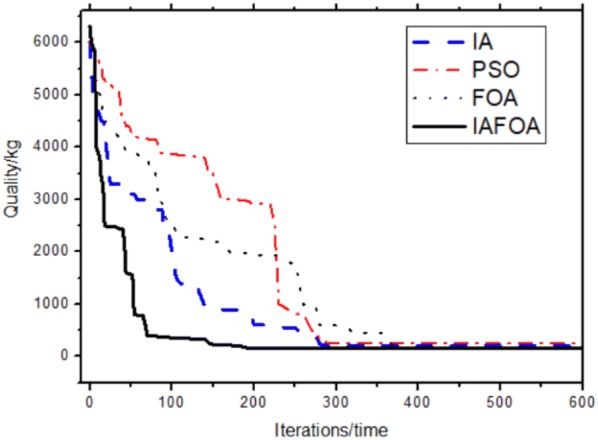
The optimization iteration curve of Example 2

The results are shown in Table 7 and Fig. 12. Under the same constraint conditions, IAFOA was used to optimize the 72-bar truss structure, and the total mass of the optimized structure was 171.98 kg. Compared to IA, mass decreased (175.03–171.98)/171.98 = 1.77%. Compared with PSO, the quality decreased (173.87–171.98)/171.98 = 1.10%; Compared with FOA, the optimization results were optimized (176.31–171.98)/171.98 = 2.52%.

**Table 7:** The classification of 200-bar truss structure

Group name	Bar number	Group name	Bar number
*A*_1_	1, 2, 3, 4	*A*_16_	82, 83, 85, 86, 88, 89, 91, 92, 103, 104, 106, 107, 109, 110, 112, 113
*A*_2_	5, 6, 7, 8, 9, 10, 12, 13, 14, 15, 16, 17	*A*_17_	115, 116, 117, 118
*A*_3_	19, 20, 21, 22, 23, 24	*A*_18_	119, 122, 125, 128, 131
*A*_4_	18, 25, 56, 63, 94, 101, 132, 170, 177	*A*_19_	133, 134, 135, 136, 137, 138
*A*_5_	26, 29, 32, 35, 38	*A*_20_	140, 143, 146, 149, 152
*A*_6_	6, 7, 9, 10, 12, 13, 15, 16, 27, 28, 30, 31, 33, 34	*A*_21_	120, 121, 123, 124, 126, 127, 129, 130, 141, 142, 144, 145, 147, 148, 150, 151
*A*_7_	39, 40, 41, 42	*A*_22_	153, 154, 155, 156
*A*_8_	43, 46, 49, 52, 55	*A*_23_	157, 160, 163, 166, 169
*A*_9_	57, 58, 59, 60, 61, 62	*A*_24_	171, 172, 173, 174, 175, 176
*A*_10_	64, 67, 70, 73, 76	*A*_25_	178, 181, 184, 187, 190
*A*_11_	44, 45, 47, 48, 50, 51, 53, 54, 65, 66, 68, 69, 71, 72, 74, 75	*A*_26_	158, 159, 161, 162, 164, 165, 167, 168, 179, 180, 182, 183, 185, 186, 188, 189
*A*_12_	77, 78, 79, 80	*A*_27_	191, 192, 193, 194
*A*_13_	81, 84, 87, 90, 93	*A*_28_	195, 197, 198, 200
*A*_14_	95, 96, 97, 98, 99, 100	*A*_29_	196, 199
*A*_15_	102, 105, 108, 111, 114

#### Example 3

**Fig. 13:**
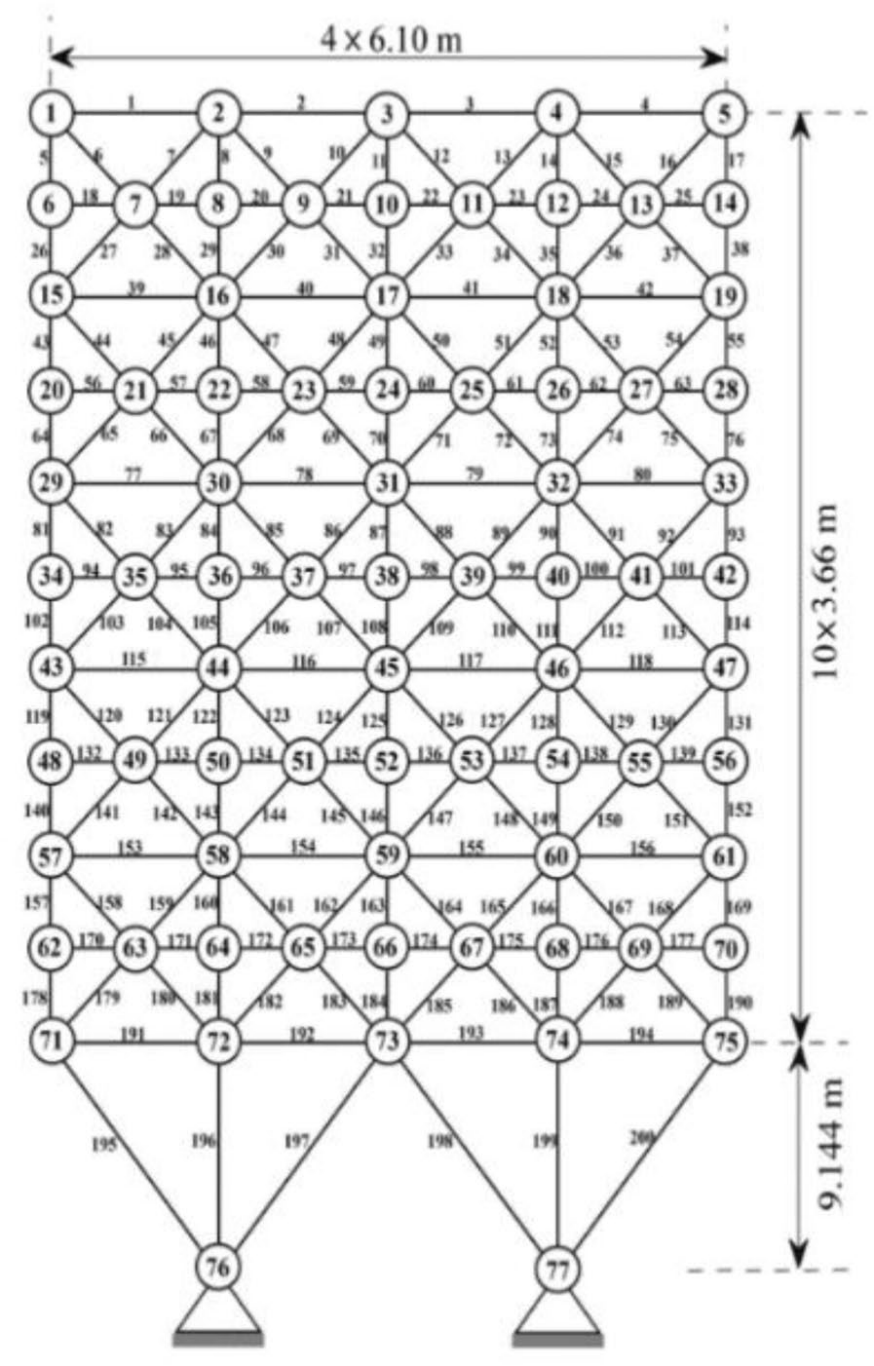
The 200-bar spatial structure

The 200-bar truss structure model [39] is established as shown in Fig. 13. 200 bars are divided into 29 groups, and it is relatively high-dimensional optimization problem. The grouping condition is shown in Table 7. Material density is $\rho = 7.86 \times 10^{3} \;{\text{kg}}/{\text{m}}^{3}$, elastic modulus is $E = 2.1 \times 10^{11} \;{\text{N}}/{\text{m}}^{2}$. The minimum permitted cross-sectional area for the truss members is taken as 0.1 cm^2^, a non-structural mass of 100 kg is attached for all free nodes.

**Fig. 14:**
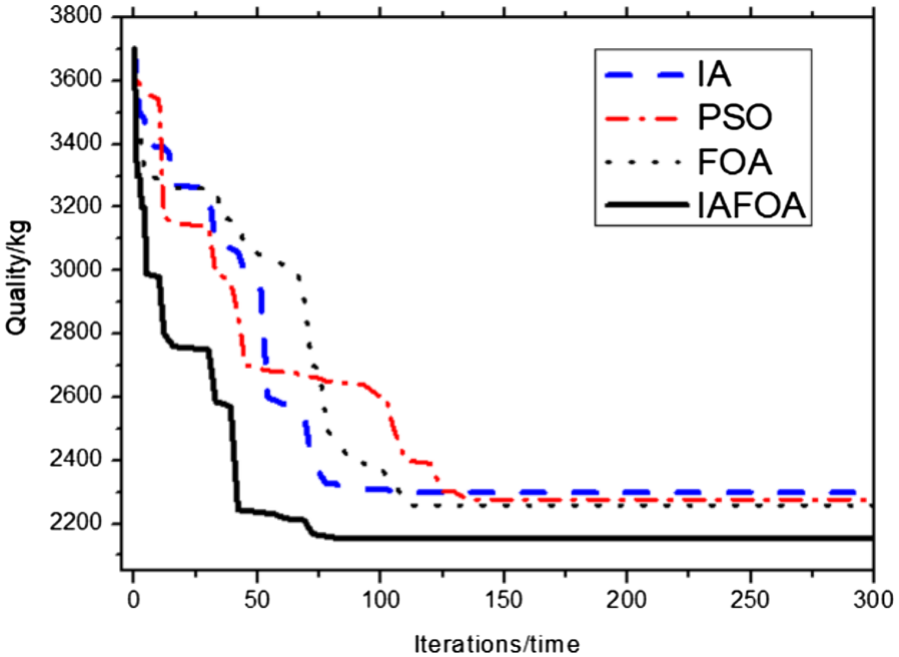
The optimization iteration curve of Example 3

The results are shown in Table 8 and Fig. 14. Under the same constraint conditions, IAFOA was used to optimize the 200-bar truss structure, and the total mass of the optimized structure was 2156.05 kg. Compared to IA, mass decreased (2298.73–2156.05)/2156.05 = 6.62%. Compared with PSO, the quality decreased (2276.59–2156.05)/2156.05 = 5.59%; compared with FOA, the optimization results were optimized (2259.86–2156.05)/2156.05 = 4.81%.

**Table 8:** Comparison of optimal designs for the 200-bar spatial truss structure

Bar group number	Bar section area (unit: m^2^)			
	IA	PSO	FOA	IAFOA
*A*_1_	0.2998	0.3078	0.4439	0.2879
*A*_2_	0.5564	0.4571	1.0438	0.4853
*A*_*3*_	0.2867	0.1006	0.3769	0.1002
*A*_4_	0.1908	0.1006	0.1494	0.1002
*A*_5_	0.8329	0.5093	0.4835	0.4985
*A*_6_	0.6598	0.8277	0.8103	0.8038
*A*_7_	0.1779	0.1051	0.2364	0.1031
*A*_8_	1.4794	1.4339	1.4554	1.3769
*A*_9_	0.4462	0.1013	1.0103	0.1000
*A*_10_	1.4566	1.5497	2.1382	1.5541
A_11_	1.2295	1.1535	0.8583	1.1507
A_12_	0.2794	0.1469	1.2718	0.1332
A_13_	1.9279	2.9492	2.0807	3.0109
A_14_	0.1179	0.1111	0.2677	0.1005
A_15_	3.4435	3.2199	3.2403	3.2584
A_16_	1.3953	1.5796	2.0098	1.6205
A_17_	0.6288	0.2901	1.5956	0.2079
A_18_	4.8937	5.1002	6.2338	5.0203
A_19_	0.6093	0.1101	0.5793	0.1328
A_20_	5.4397	5.4594	3.0520	4.8531
A_21_	1.8439	2.9007	1.8121	2.0138
A_22_	1.4273	0.6489	1.2986	0.7201
A_23_	8.1702	7.6592	5.8810	7.7219
A_24_	0.3271	0.1379	0.2324	0.1819
A_25_	10.902	8.0097	7.7536	8.9438
A_26_	2.9359	2.7602	2.6871	2,9953
A_27_	19.5329	10.4765	10.2694	10.2049
A_28_	20.9768	21.3501	21.5704	20.6507
A_29_	15.7301	10.5014	8.2901	11.5468
Weight (kg)	2298.73	2276.59	2259.86	2156.05

## Conclusion

With the development of civil engineering, the structural optimization is more and more important. To find a new method for the truss structure optimization, an improved FOA was proposed. To overcome the shortage of the basic FOA, the IA was introduced into FOA, and a new IAFOA algorithm was proposed. Combining the global search capability of the original standard FOA algorithm and the strong local search capability of IA algorithm itself, the IAFOA achieves good results in numerical simulation with high ability of solving optimization problems. Although these improvements can overcome some shortcomings of the algorithm, it lacks the adjustment of parameter stagnation step number wide value *T* and fixed probability $P^{*}$. In the future, more detailed improvements are needed to make IAFOA algorithm converge faster and more accurately. In addition, the example in this paper is relatively simple, and the application of the algorithm in the optimization of complex structures needs to be further studied.

## Data Availability

All data generated or analyzed during this study are included in this published article.
